# The profile of suspected criminal offenders referred for psychiatric evaluation on an outpatient basis at Ngwelezana Hospital

**DOI:** 10.4102/sajpsychiatry.v27i0.1722

**Published:** 2021-07-29

**Authors:** Sithembisile Mngadi, Andrew Tomita, Vusi Khanyile, Bonginkosi Chiliza

**Affiliations:** 1 Department of Psychiatry, Nelson R. Mandela School of Medicine, University of KwaZulu-Natal, Durban, South Africa; 2 KwaZulu-Natal Research Innovation and Sequencing Platform (KRISP), College of Health Sciences, University of KwaZulu-Natal, Durban, South Africa; 3 Department of Psychiatry, Madadeni Provincial Hospital, Newcastle, South Africa

**Keywords:** outpatient forensic assessment, fitness to stand trial assessment, capacity assessment, long waiting list for pretrial assessment, pretrial detention

## Abstract

**Background:**

Some suspected criminal offenders in South Africa are required to undergo forensic psychiatry assessments before or during the trial, which can be delayed as a result of the shortage of psychiatrists and inpatient forensic psychiatry beds. In KwaZulu-Natal (KZN) province, only one hospital (Fort Napier Hospital [FNH]) is designated for the 30-day inpatient forensic psychiatry assessments and there is a long waiting list for suspected criminal offenders awaiting assessment. There is a need to find ways of alleviating the backlog in the waiting list, with the use of outpatient forensic assessments being a possible adjunctive method.

**Aim:**

To determine the demographic, clinical and forensic profile of suspected criminal offenders referred for outpatient preliminary assessment to Ngwelezana Hospital, and identify the profile of those who most likely require referral to FNH for a 30-day inpatient assessment.

**Setting:**

The study was conducted at Ngwelezana Tertiary Hospital, in KZN, South Africa.

**Methods:**

We conducted a retrospective chart review of 207 suspected criminal offenders referred for outpatient forensic assessment from January 2009 to June 2015.

**Results:**

The majority of the participants were males (94.2%), with a diagnosis of substance use disorder (28.2%), intellectual disability (23.4%) or psychotic disorders (21.8%). Forty three per cent were charged with sexual crimes and 10.7% with murder. Fifty seven per cent were recommended for referral to FNH for a 30-day inpatient forensic assessment, whilst 43% were not recommended for referral. Those recommended for inpatient assessment were significantly more likely to have a lower level of education (*p* = 0.02), to be on a disability grant (*p* < 0.01), and to have been diagnosed with intellectual disability (*p* < 0.01), than those not recommended for referral.

**Conclusion:**

Identifying the characteristics of suspected criminal offenders who are most likely to be recommended for referral to FNH will potentially reduce the number of unnecessary referrals.

## Background

Some criminal cases in South Africa require a forensic psychiatric assessment if, during any stage of the court proceedings, there is evidence to suggest that the suspected criminal offender is incapable of understanding criminal proceedings (by reason of mental illness or intellectual disability) in order to make a proper defence.^1^

Suspected adult criminal offenders are referred for psychiatric assessment in accordance with the *Criminal Procedure Act* (CPA) 51 of 1977, sections 77, 78, and 79,^2^ whilst children are referred in accordance with the *Child Justice Act* (CJA) 75 of 2008 Chapter 2(ss 7–11).^3^

Section 77 of the CPA refers to the capacity of the accused to understand court proceedings, section 78 to the presence of mental illness or intellectual disability and criminal responsibility,^1^ section 79 (b) refers to the panel (at least two psychiatrists) for the purposes of enquiry and report under both sections.^2^ Section 79 (b) states that where the accused is charged with murder, culpable homicide, rape or compelled rape, or any other charge involving serious violence, the relevant enquiry shall be conducted and reported upon by the head of the designated health establishment or the psychiatrist appointed by him or her, the psychiatrist appointed by the court and a clinical psychologist at the discretion of the court. In the case of children, the CJA refers to proof of criminal capacity.^3^ The process of a 30-day inpatient assessment involves a multidisciplinary team that is involved in conducting the assessment of the referred suspected criminal offender. However, with no profiling of those who are more likely to require such assessments being available, to reduce the number of suspected criminal offenders being referred for a 30-day inpatient assessment, the problem of long waiting lists is likely to continue.

Whilst Forensic Psychiatry is now a recognised subspecialty by the Health Professions Council of South Africa (HPCSA), having been gazetted on 15 April 2011,^4^ it is faced with a number of challenges. The shortage of forensic psychiatrists and public sector inpatient forensic observation facilities has resulted in long waiting lists for forensic psychiatric assessments.^5^ The process of a 30-day inpatient assessment is labour intensive, time consuming and expensive.^6,7^ The Department of Health’s turnaround strategy to reduce the long waiting list found that it was legally compatible for psychiatric forensic observations to be conducted by only one psychiatrist in correctional centres, or on an outpatient basis in specific health establishments.^5^ As of March 2010, single psychiatrist observations were conducted in the Northern Cape, North West, Limpopo and the Free State provinces,^5^ which were helpful in reducing the number of awaiting-trial detainees requiring forensic assessment, as everyone did not require 30-day inpatient assessments.^5^

Few studies have reported on such assessments in South Africa, with most assessments being carried out on an inpatient basis,^8,9,10^ those being conducted on a preliminary outpatient basis having taken place in rural, under-resourced settings.^6,11^ A number of countries have explored the option of outpatient forensic observations, including Israel, where Olmer found that in 65.2% of the court-ordered psychiatric assessments, the assessing psychiatrist felt that there was no need for hospitalisation or inpatient forensic assessment. The author contended that an outpatient unit dedicated to writing these reports could significantly reduce the rates of hospitalisation.^12^ There is also evidence to support this practice in Canada, where Roesch et al. reported that 88% of the court orders for evaluation were still being referred to inpatient services, leaving only 12% to be assessed as outpatients.^13^ They argued that inpatient assessments are unnecessary for all but perhaps a small percentage of cases, as most determinants of competency can easily be made on the basis of brief screening interviews.^13^

Grisso et al. in a study from the United States of America (USA) noted the following competence evaluation system methods:

- ‘A traditional model where pre-trial evaluations are conducted in inpatient settings.
- The private practitioner model, where outpatient assessments are conducted by practitioners in the community.
- A community-based system, the assessments take place at the local outpatient mental health facilities or court clinics.
- Modified traditional, assessments are carried out at centralised mental health facilities on an outpatient basis and,
- The Mixed model.’^14^

Grisso et al. concluded that only a few states use inpatient facilities for such assessments, having been removed mainly because of the high associated costs.^14^ This study therefore aimed to describe the demographic, clinical and forensic profile of suspected criminal offenders referred for outpatient preliminary evaluation to Ngwelezana Hospital, KwaZulu-Natal (KZN) province, South Africa. Fort Napier Hospital (FNH) is the only facility in KZN designated to conduct 30-day inpatient forensic psychiatry assessments and as a result is burdened with all the referrals from the courts across the province for these assessments.

## Methodology

### Study design

A descriptive study design was used to conduct a retrospective assessment of all records of adults and children referred by the courts for outpatient forensic evaluation by the specialist psychiatrists working at Ngwelezana Hospital from January 2009 to June 2015.

### Study setting

Ngwelezana Hospital is situated in the town of Empangeni, and is designated as a tertiary hospital for King Cetshwayo, Zululand and Umkhanyakude districts. It is the only hospital in uMhlathuze sub-district functioning as a level I (district), level II (regional) and level III (provincial tertiary) facility.^15^

### Sample

During the study period, 387 files were retrieved from the outpatient clinic that related to suspected criminal offenders, of which 180 were excluded: 81 files belonged to victims of crime assessments, 21 were general psychiatry files, 6 were empty and 19 were without reports. Others that were excluded consisted of witness assessment files (4), medical boarding (2), return to work assessment (1), assessment for custody (2), concern of abuse or neglect of minors (1), referral for assessment but not conducted (1) and a medical file (1). In addition, 41 files had to be excluded because of no information being provided regarding recommendations, which resulted in the files of 207 suspected criminal offenders being included as participants.

### Data collection method

The assessment records of suspected criminal offenders that were assessed for court purposes at Ngwelezana Hospital were retrieved from the Psychiatry department. Data were collected manually from files using a standardised data collection sheet designed by the primary investigator. A study number was allocated for every file, with a list – that was only accessible to the primary investigator – being kept linking the study number to the files. All data were coded to prevent participant identification, and entered into an anonymised Microsoft Excel spreadsheet for analysis.

### Statistical analysis

Three analyses were conducted, the first being descriptive statistics to summarise the socio-demographic, clinical and forensic profiles of the participants. In the second analysis, their profiles (socio-demographic, clinical and forensic) were compared against the likelihood of being referred to FNH based on a chi-square test. Lastly, eight logistic regression models were fitted to investigate the interaction between crime and clinical diagnosis. The interpretation of interaction between categorical variables was difficult, with the post-estimation margins on the predicted probability of outcome (referral to FNH) based on crime and clinical diagnosis being calculated, all data analysis being done using the STATA 16 statistical software.

### Ethical considerations

Ethical approval was granted by the University of KwaZulu-Natal Biomedical Research Ethics Committee (reference number: BE 420/15). Permission to conduct the study was obtained from the KwaZulu-Natal Department of Health and the Ngwelezana Hospital Management.

## Results

### Demographic profile

Table 1 presents a summary of the socio-demographic, clinical and forensic factors, with a majority of the 207 participants being male (*n* = 195, 94.2%), 170 (84.6%) being adults over 18 years and 31 (15.4%) being children. The majority were single (*n* = 201, 97.6%), had primary school education (*n* = 102, 50.5%) and were unemployed (*n* = 152, 75.2%) at the time of arrest. A small number of participants were attending special schools for intellectually disabled children (*n* = 4, 2.0%), and receiving a disability grant (*n* = 15, 7.25%).

**TABLE 1:** Socio-demographic, clinical and forensic profiles of overall sample.

Variables	Characteristics	*n*	%
Gender	Male	195	94.2
	Female	12	5.8
Age category	< 18	31	15.4
	18–35	125	62.2
	36–50	31	15.4
	50+	14	7.0
Marital status	Single	201	97.6
	Married	4	1.9
	Widowed	1	0.5
Educational attainment	None	18	8.9
	Primary	102	50.5
	Secondary	61	30.2
	Grade 12	17	8.4
	Special needs	4	2.0
Unemployed	No	50	24.8
	Yes	152	75.2
Disability grant	No	192	92.8
	Yes	15	7.2
**Diagnosis**			
Intellectual disability	No	154	76.6
	Yes	47	23.4
Psychosis	No	158	78.2
	Yes	44	21.8
Substance use disorder	No	145	71.8
	Yes	57	28.2
**Charge**			
Murder	No	183	89.3
	Yes	22	10.7
Rape	No	116	56.9
	Yes	88	43.1
Assault	No	172	84.3
	Yes	32	15.7
Theft	No	183	89.3
	Yes	22	10.7

### Clinical profile

Out of the 207 participants, 57 (28.2%) had a substance use disorder, 47 (23.4%) an intellectual disability, 44 (21.8%) a psychotic disorder; malingering could not be excluded in 29 (14%) and 45 (22.0%) had other diagnoses.

### Forensic information

Eighty-eight (43.1%) participants were charged with sexual offenses including rape and sexual assault, 32 (15.7%) with assault (common assault and assault with intent to cause grievous bodily harm), 22 (10.7%) with murder and the same number with theft. The number recommended for referral to FNH for a full inpatient assessment according to the CPA sections 77 and 78 was 117 (57.4%). This resulted in 87(42.7%) not being recommended for referral to FNH and being given other recommendations, such as referral to psychologist, involuntary treatment, substance abuse rehabilitation centre and referral back to the court for continuation of court proceedings.

### Comparison of socio-demographic profiles of those referred and not referred to Fort Napier Hospital

The participants who were recommended for referral to FNH were significantly more likely to be on a disability grant compared to those not referred, (12.0% vs 1.1%: *p* < 0.01) (Table 2), and also significantly more likely to have had a lower level of education (*p* = 0.02).

**TABLE 2:** Socio-demographic profile comparison between group referred to Fort Napier Hospital and group not referred.

Characteristics	Not referred		Referred		Total	*df*	χ^2^	*p*
	*n*	%	*n*	%	*n*			
**Gender**	-	-	-	-	-	1	3.01	0.08
Male	79	90.8	113	96.6	192	-	-	-
Female	8	9.2	4	3.4	12	-	-	-
**Age category**	-	-	-	-	-	3	4.49	0.21
< 18	14	16.5	15	13.3	29	-	-	-
18–35	58	68.2	67	59.3	125	-	-	-
36–50	8	9.4	22	19.5	30	-	-	-
50+	5	5.9	9	8.0	14	-	-	-
**Educational attainment**	-	-	-	-	-	3	9.87	0.02
None or special needs	5	5.8	17	15.0	22	-	-	-
Primary	39	45.3	63	55.8	102	-	-	-
Secondary	33	38.4	25	22.1	58	-	-	-
Grade 12	9	10.5	8	7.1	17	-	-	-
**Unemployed**	-	-	-	-	-	1	1.22	0.27
No	24	28.6	25	21.7	49	-	-	-
Yes	60	71.4	90	78.3	150	-	-	-
**Disability grant**	-	-	-	-	-	1	8.57	< 0.01
No	86	98.9	103	88.0	189	-	-	-
Yes	1	1.1	14	12.0	15	-	-	-

Analysis for marital status was not included given overwhelming majority were single.

### Comparison of clinical profiles between those referred and not referred to Fort Napier Hospital

More participants who were recommended for referral to FNH had intellectual disability than those who were not referred (76.6% vs. 23.4%, *p* < 0.01) (Table 3). There was also a significant association between a diagnosis of substance use disorder and *not* being referred to FNH (64.9% vs. 35.1%, *p* < 0.01).

**TABLE 3:** Clinical diagnosis comparison between group referred to Fort Napier Hospital and group not referred.

Variables	Not referred		Referred		Total	*df*	*χ*^2^	*p*
	*n*	%	*n*	%	*n*			
**Diagnosis: Intellectual disability**	-	-	-	-	-	1	9.63	< 0.01
No	75	49.0	78	51.0	153	-	-	-
Yes	11	23.4	36	76.6	47	-	-	-
**Diagnosis: Psychosis**	-	-	-	-	-	1	2.07	0.15
No	63	40.1	94	59.9	157	-	-	-
Yes	23	52.3	21	47.7	44	-	-	-
**Diagnosis: Substance use disorder**	-	-	-	-	-	1	15.92	< 0.01
No	49	34.0	95	66.0	144	-	-	-
Yes	37	64.9	20	35.1	57	-	-	-

### Comparison of forensic profiles between those referred and not referred to Fort Napier Hospital

The participants charged with serious crimes were significantly more likely to be recommended for referral to FNH, for example rape (79.3%, *p* < 0.01) and murder (86.4%, *p* < 0.01) (Table 4). Those charged with less serious crimes were significantly more likely *not* to be referred to FNH, for example assault (74.2% vs. 25.8%, *p* < 0.01) and theft (76.2% vs. 23.8%, *p* < 0.01).

**TABLE 4:** Forensic profile comparison between group referred to Fort Napier Hospital and group not referred.

Variables	Not referred		Referred		Total	*df*	*χ*^2^	*p*
	*n*	%	*n*	%	*n*			
**Charge: Murder**	-	-	-	-	-	1	8.46	< 0.01
No	83	46.1	97	53.9	180	-	-	-
Yes	3	13.6	19	86.4	22	-	-	-
**Charge: Rape**	-	-	-	-	-	1	30.60	< 0.01
No	68	59.6	46	40.4	114	-	-	-
Yes	18	20.7	69	79.3	87	-	-	-
**Charge: Assault**	-	-	-	-	-	1	14.77	< 0.01
No	63	37.1	107	62.9	170	-	-	-
Yes	23	74.2	8	25.8	31	-	-	-
**Charge: Theft**	-	-	-	-	-	1	10.83	< 0.01
No	70	38.7	111	61.3	181	-	-	-
Yes	16	76.2	5	23.8	21	-	-	-

### Predicted probability of outcome (referral to Fort Napier Hospital recommendation) based on crime and clinical diagnosis

There was a higher probability of being recommended for referral to FNH in participants who had been charged with rape and suffered from intellectual disability compared to those who did not have a combination of these findings (Table 5). This was also the case in the absence of psychosis and other criminal charges, such as theft and assault, and confirms the significance of rape and intellectual disability in warranting referral to FNH.

**TABLE 5:** Predicted probability of outcome (referral to Fort Napier Hospital recommendation) based on crime and clinical diagnosis.

Variables	Predicted probability	Standard error	95% CI	
**Rape#Intellectual disability**				
No#No	0.41	0.05	0.31	0.50
No#Yes	0.33	0.16	0.03	0.64
Yes#No	0.73	0.06	0.60	0.85
Yes#Yes	0.86	0.06	0.75	0.98
**Assault#Intellectual disability**				
No#No	0.57	0.04	0.49	0.66
No#Yes	0.79	0.06	0.66	0.91
Yes#No	0.25	0.08	0.09	0.41
Yes#Yes	0.33	0.27	-0.20	0.87
**Rape#Psychosis**				
No#No	0.37	0.05	0.26	0.47
No#Yes	0.48	0.09	0.31	0.66
Yes#No	0.83	0.04	0.74	0.91
Yes#Yes	0.50	0.16	0.19	0.81
**Assault#Psychosis**				
No#No	0.66	0.04	0.58	0.74
No#Yes	0.50	0.09	0.33	0.67
Yes#No	0.18	0.08	0.02	0.34
Yes#Yes	0.44	0.17	0.12	0.77
**Theft#Psychosis**				
No#No	0.64	0.04	0.56	0.72
No#Yes	0.50	0.08	0.35	0.65
Yes#No	0.19	0.10	0.00	0.38
Yes#Yes	0.33	0.27	-0.20	0.87
**Rape#Substance use disorder**				
No#No	0.48	0.06	0.36	0.61
No#Yes	0.28	0.07	0.15	0.41
Yes#No	0.80	0.05	0.71	0.89
Yes#Yes	0.70	0.14	0.42	0.98
**Assault#Substance use disorder**				
No#No	0.69	0.04	0.61	0.77
No#Yes	0.44	0.08	0.29	0.59
Yes#No	0.39	0.11	0.16	0.61
Yes#Yes	0.08	0.07	-0.07	0.22
**Theft#Substance use disorder**				
No#No	0.69	0.04	0.62	0.77
No#Yes	0.39	0.07	0.25	0.52
Yes#No	0.25	0.13	0.01	0.49
Yes#Yes	0.14	0.13	-0.12	0.40

CI, confidence interval.

## Discussion

This study was a retrospective chart review of preliminary outpatient assessments to establish the profile of suspected criminal offenders assessed at Ngwelezana Hospital. Of the 207 participants, 43% were not recommended for referral to FNH. Given the resource challenges facing South Africa’s public health services and the long waiting lists for forensic assessments, outpatient forensic assessments could serve as a possible adjunctive to the current 30-day inpatient method.

### Demographic profile

The majority of suspected criminal offenders assessed in this study were male (95.65%), single, unemployed and had a lower level of education, which is similar to other local outpatient and inpatient studies.^9,10,11,16^ The median age in this study was 29 years, similar to other studies; for example, Zapf et al.’s study found a median age of 33 years,^17^ Calitz et al. of 30 years^16^ and Marais et al. of 32 years.^8^ The demographic profiles seen on an outpatient basis appears to be in keeping with those observed on an inpatient basis in both local and international studies.

### Forensic profile

The majority of the suspected offenders were charged with offenses against persons, including those of a sexual nature, murder and attempted murder. Individuals charged with serious crimes were significantly more likely to be recommended for referral to FNH for a 30-day inpatient observation than those charged with less serious crimes. In a Canadian study, Whittemore also found that the majority of participants (67.1%) were charged with crimes against persons.^18^

### Clinical profile

The high rate of substance use disorders (27.53%) found in this study was not in keeping with the literature, where schizophrenia or psychotic disorders are reported to be the most common diagnosis.^8,9,18^ This may also be reflective of regional patterns of substance use in the studied population, and highlights the lack of appropriate facilities to address the problem of substance abuse in communities. The impact of substance abuse on offending behaviour is well documented in the literature.^19,20,21,22^ The majority of suspected criminal offenders who were recommended for referral to FNH had a diagnosis of intellectual disability (22.7%), which is in keeping with an observation made in a local study (36.26%) by Houdi.^10^

The evaluation of competence and culpability (criminal responsibility) in individuals with intellectual disability is extremely important, yet difficult, because of the cognitive deficits and limited problem-solving skills.^23^ The assessment of these individuals must take into account the range of social, cognitive, behavioural and communicative characteristics of the assessee.^24^ Studies in KZN found a higher prevalence of individuals with intellectual disability (22.7% in this study and 36.26% in the study by Houdi^10^) compared with studies in other provinces (19% in the study by Ndala^11^ and 20.1% in the study by Du Plessis^9^). This indicates a gap in addressing the needs of this population in communities to prevent high levels of offending behaviour, as well as the proper diversion and rehabilitation of individuals with intellectual disability.

Outpatient forensic psychiatric assessments should be considered as an option across South Africa to assist with reducing the number (43% in this study) of suspected criminal offenders from being unnecessarily referred for a 30-day inpatient observation and delaying finalisation of their cases.

### Forensic profiles between those referred and not referred to Fort Napier Hospital

The long waiting lists for forensic observations have a considerable impact on the administration of justice in South Africa, which has seen a backlog of approximately 30 000 criminal cases over the last few years.^25^ Due to the backlog in criminal cases in South Africa, many suspected criminal offenders awaiting trial wait for months for fitness to stand trial assessments,^26^ during which time they often remain under poor conditions that infringe a number of their human rights. Restriction of liberty is a notable infringement, with the *Constitution of South Africa*, section 12, stating that ‘[*e*]veryone has the right to freedom and security of the person, and the right not to be deprived of freedom arbitrarily or without just cause’.^27^ Disruption of family life and loss of employment are some of the consequences endured by these individuals.^28^ The South African Society Of Psychiatrists (SASOP) position statement in 2012 noted that there were 10 units in the country that were designated to conducting forensic psychiatric assessments. However, these are unevenly distributed across the nine South African provinces, with some having more than one whilst others have none,^29^ further crippling the efficient processing of psychiatric forensic assessments.

In many countries, including South Africa, people in pretrial detention are held in overcrowded and insanitary conditions, at risk of mistreatment and violence.^28^ These pretrial detainees usually have no access to rehabilitative programmes, and they are often detained in worse conditions than those of sentenced offenders.^28^ The time spent in prison delays meaningful interventions at community level to address the reasons for their offending behaviour, such as substance abuse or mental illness.^28^ In addition, pretrial detainees with untreated mental illness are at significant risk of injury and victimisation, and even death by suicide.^30^

Approximately 20 years ago, Canada (Ontario province) experienced similar challenges to those currently faced by South Africa, with the time between court-ordered and inpatient assessment being lengthy, resulting in the accused suffering from a mental illness going untreated, and those without mental illness experiencing a prolonged incarceration and restricted liberty.^31^ A pilot study in Canada demonstrated significant cost-saving measures when inpatient evaluation costs were compared with running outpatient fitness to stand trial clinics, in detention centres.^31^ Zapf and Roesch assert that the use of screening tools, for example the Fitness Interview Test-Revised (FIT-R), could help facilitate fitness to stand trial assessments at community level rather than depend on traditional inpatient forensic assessments.^17^

Gowensmith noted that the USA, like South Africa, experienced challenges with competency or fitness to stand trial assessments, with long waiting lists and shortage of evaluators.^25^ In the USA, orders for fitness to stand trial have risen sharply over the past decade (206% increase in Colorado),^32^ with approximately 60 000 of these evaluations being ordered annually.^33^ To address this growing need, there has been a gradual shift over the last few years to most assessments now taking place in county jails or in community-based settings instead of hospitals,^7^ with a resulting significant reduction in costs.^25^

Pillay discussed a number of challenges that are relevant to the South African context and amongst other recommendations noted the use of outpatient assessments as an alternative to the 30-day observation.^26^ In a Limpopo province study, Ndala stated that more than 80% of forensic assessments were carried out on an outpatient basis.^11^ A South African study by Calitz et al. supported the use of a single method rating and found it to be as reliable as the multidisciplinary team in evaluating fitness to stand trial. The authors found this single method rating to be cost-effective and noted that it avoided unnecessary inpatient assessments.^16^ The practice of outpatient forensic assessments would therefore allow those with less serious crimes to be processed quickly and efficiently, without causing unnecessary delays and consuming much fewer resources than during a 30-day inpatient assessment.

### Recommendations

It is recommended that individuals accused of violent crimes be referred straight to FNH for an inpatient forensic assessment, as they would require a panel and multidisciplinary assessment (section 79 of CPA) because of the seriousness of their criminal charges. Considering the available evidence, the use of outpatient, community settings can play a significant role in reducing the backlog in the waiting list of pretrial forensic assessments.

### Limitations of the study

This study indicated the value of these outpatient preliminary assessments, although the results are not generalisable to other settings because of the small sample size, with only one rural site being assessed and many files being excluded because of insufficient information. This was a retrospective review of records, with the quality of the evidence collected depending on what was available in the clinical files. There is limited published evidence on outpatient preliminary forensic psychiatry assessments in South Africa, which resulted in most of the studies used having been done on inpatient populations. There is therefore a need for further studies to be conducted in other settings within South Africa to ensure population specific and relevant interventions.

## Conclusion

Conclusions that can be drawn from this study are that suspected criminal offenders requiring forensic psychiatry assessment should be directly referred for inpatient forensic assessment if they have committed a serious or violent crime as stipulated in the CPA, are diagnosed with intellectual disability or are receiving a disability grant. Those who have been charged with theft and assault can be referred for outpatient forensic psychiatry assessment by one psychiatrist. Those requiring a panel and multidisciplinary assessment should be referred for inpatient forensic assessment, whilst single psychiatrist assessments can be carried out on an outpatient basis, to ease the backlog of long waiting lists for forensic psychiatry assessments.

## References

[1] Government of South Africa. Criminal Procedure Amendment Act [No. 4 of 2017]. [homepage on the internet]. [cited 2020 Jun 8]. Available from https://www.gov.za/documents/criminal-procedure-act-4-2017-english-afrikaans-29-jun-2017-0000

[2] Africa RoS. Criminal Procedure Act 51 of 1977. Pretoria: Juta Law; 2008.

[3] Africa RoS. Child Justice Act No. 75 of 2008. Pretoria: Government Gazette; 2008.

[4] Department of Health. Government notice no. R. 341, Regulations Gazette no. 34205, Government Gazette, Regulation 3, 15 April 2011. Cape Town: Government Printing Works.

[5] Dept of Health Republic of South Africa. A turnaround strategy to reduce the long waiting list and waiting period for forensic psychiatric observations/evaluations of awaiting trial detainees referred by courts in terms of section 77,78 & 79 of the Criminal Procedure Act no 51 of 1977. Cape Town: Juta, 2010; p. 1–14.

[6] Meyer S, Slabber M, Van Rensburg P, Nel M. Use of the Judicial Section 9 certification in the Free State. S Afr J Psychiatr. 2004;10(4):104–108. 10.4102/sajpsychiatry.v10i4.122

[7] Gowensmith WN, Murrie DM, Packer IK. Forensic mental health consultant review final report (Contract No. 1334–91698). Vancouver, WA: Washington State Department of Social and Health Services; 2014.

[8] Marais B, Subramaney U. Forensic state patients at Sterkfontein Hospital: A 3-year follow-up study. S Afr J Psychiatr. 2015;21(3):86–92. 10.4102/sajpsychiatry.v21i3.708

[9] Du Plessis ED, Du Plessis HJ, Nel HC, et al. Accountable or not accountable: A profile comparison of alleged offenders referred to the Free State Psychiatric Complex Forensic Observation Ward in Bloemfontein from 2009 to 2012. S Afr J Psychiatr. 2017;23:1054. 10.4102/sajpsychiatry.v23i0.1054 30263191 PMC6138100

[10] Houidi A, Paruk S. Profile of forensic state patients at a psychiatric unit in KwaZulu Natal, South Africa: Demographic, clinical and forensic factors. J Forensic Psychiatr Psychol. 2018;29(4):544–556. 10.1080/14789949.2018.1425471

[11] Ndala M. An analysis of offenders referred for forensic observation in Limpopo from January 2005 to December 2006. Pretoria: University of Limpopo (Medunsa Campus); 2009.

[12] Olmer A, Greenberg B, Strous RD. Assessing the Need for hospitalization in order to conduct a psychiatric evaluation as part of criminal law procedure. Isr Med Assoc J. 2015;17(9):533–537. 26625540

[13] Roesch R, Zapf PA, Golding SL, Skeem JL. Defining and assessing competency to stand trial. In: Hess AK, Weiner IB, editors. The handbook of forensic psychology. 2nd ed. New York: John Wiley & Sons, Inc., 1999; p. 327–349.

[14] Grisso T, Cocozza JJ, Steadman HJ, Fisher WH, Greer A. The organization of pretrial forensic evaluation services: A rational profile. Law Hum Behav. 1994;18(4):377–393. 10.1007/BF01499046

[15] KwaZulu-Natal Department of Health. Ngwelezana Hospital. [homepage on the internet]. [cited 2020 Oct 10]. Available from: http://www.kznhealth.gov.za/ngwelezanahospital.htm

[16] Calitz FJW, Van Rensburg PHJJ, Oosthuizen H, Verschoor T. Criteria for fitness to stand trial criminal trial. S Afr Med J. 1996;86(6):734–737. 9180765

[17] Zapf PA, Roesch R. Assessing fitness to stand trial: A comparison of institution-based evaluations and a brief screening interview. Can J Community Ment Health. 1997;16:53–66.

[18] Whittemore KE, Ogloff JRP, Roesch R. An investigation of competency to participate in legal proceedings in Canada. Can J Psychiatr. 1997;42(8):869–875. 10.1177/070674379704200811 9356777

[19] Boles SM, Miotto K. Substance abuse and violence: A review of the literature. Aggression Violent Behav. 2003;8(2):155–174. 10.1016/S1359-1789(01)00057-X

[20] Pulay AJ, Dawson D, Hasin DS, et al. Violent behavior and DSM-IV psychiatric disorders: Results from the national epidemiologic survey on alcohol and related conditions. J Clin Psychiatr. 2008;69(1):12–22. 10.4088/JCP.v69n0103 PMC2922980 18312033

[21] Bennett T, Holloway K. The causal connection between drug misuse and crime. Br J Criminol. 2009;49(4):513–531. 10.1093/bjc/azp014

[22] Boden JM, Ferusson DM, Horwood LJ. Alcohol misuse and violent behavior: Findings from a 30-year longitudinal study. Drug Alcohol Depend. 2012;122(1–2):135–141. 10.1016/j.drugalcdep.2011.09.023 22015176

[23] Jones J. Persons with intellectual disabilities in the criminal justice system: Review of issues. Int J Offender Ther Comp Criminol. 2007;51(6):723–733. 10.1177/0306624X07299343 17636203

[24] Kaliski SZ. Psycholegal assessment in South Africa. New York: Oxford University Press; 2006.

[25] Gowensmith WN, Robinson KP. Fitness to stand trial evaluation challenges in the United States: Some comparisons with South Africa. S Afr J Psychol. 2017;47(2):148–158. 10.1177/0081246316673523

[26] Pillay AL. Competency to stand trial and criminal responsibility examinations: Are there solutions to the extensive waitng list? S Afr J Psychol. 2014;44(1):48–59. 10.1177/0081246313516263

[27] Government of South Africa. Constitution of the Republic of South Africa [No. 108 of 1996] [homepage on the Internet]. [cited 2020 Jun 8]. Available from: https://www.gov.za/sites/default/files/images/a108-96.pdf

[28] Heard C, Fair H. Pre-trial detention and its over use: Evidence from ten countries. Institute for crime and justice policy research [homepage on the Internet]. [cited 2020 Jun 8]. Available from: http://www.prisonstudies.org/world-prison-brief

[29] Van Rensburg BJ. The South African Journal of Psychiatrists (SASOP) and SASOP State Employed Special Interest Group (SESIG) position statements on psychiatric care in the public sector. S Afr J Psychiatr. 2012;18(3):16. 10.4102/sajpsychiatry.v18i3.374

[30] Hayes LM. Prison suicide: An overview and a guide to prevention. Prison J. 1995;75(4):431–456. 10.1177/0032855595075004003

[31] Chaimowitz GA, Ferencz J. Cost savings associated with fitness-to-stand trial assessments in detention centres: A pilot program. Can J Psychiatr. 1999;44(8):808–810. 10.1177/070674379904400809 10566113

[32] Steffen J. Colorado sees sharp rise in competency exams for criminal defendants [homepage on the Internet]. Denver Post. 2014, 4 19. [cited 2020 Jun 10]. Available from: http://www.denverpost.com/2014/04/19/colorado-sees-sharp-rise-in-competencyexams-for-criminal-defendants/

[33] Melton GB, Petrila J, Poythress NG, Slobogin C. Psychological evaluations for the courts: A handbook for mental health professionals and lawyers. 3rd ed. New York, NY: Guilford Press; 2007.

